# Supplementary material for: Polyketide synthase gene clusters in the ericoid mycorrhizal fungus Oidiodendron maius: genome-wide prediction, expression profiling, and functional characterization of knockout mutants
Source: Front Plant Sci. 2026 Sep 16;17:1924430. doi: 10.3389/fpls.2026.1924430 (PMC13623903; doi:10.3389/fpls.2026.1924430)
Supplement: Supplementary file 1 [file SupplementaryFile1.zip › Supplementary Materials/Supplementary Folder 1.zip!/Oidma1_AssemblyScaffolds_Repeatmasked/knownclusterblast/cluster14/jgi.p_Oidma1_178014_mibig_hits.html]

| MIBiG Protein | Description | MIBiG Cluster | MiBiG Product | % ID | % Coverage | BLAST Score | E-value |
| --- | --- | --- | --- | --- | --- | --- | --- |
| AAN59954.1 | unknown | BGC0001258 | 1,8-dihydroxynaphthalene\_biosynthetic\_gene\_cluster | 40 | 47.3076923077 | 76 | 5.4e-14 |
| AAS90003.1 | AflR | BGC0000007 | Aflatoxin\_biosynthetic\_gene\_cluster | 34 | 41.1538461538 | 55 | 1e-07 |
| ANF07281.1 | C6\_transcription\_factor | BGC0001385 | Byssochlamic\_acid\_/\_agnestadrides\_biosynthetic\_gene\_cluster | 28 | 51.5384615385 | 54 | 2.9e-07 |
| BAE71318.1 | regulatory\_protein | BGC0000004 | Aflatoxin\_biosynthetic\_gene\_cluster | 31 | 45.3846153846 | 52 | 6.5e-07 |
| AAS90091.1 | AflR | BGC0000006 | Aflatoxin\_biosynthetic\_gene\_cluster | 31 | 45.3846153846 | 52 | 6.5e-07 |
| AAS90026.1 | AflR | BGC0000008 | Aflatoxin\_biosynthetic\_gene\_cluster | 31 | 45.3846153846 | 52 | 6.5e-07 |
| AAS90086.1 | AflR | BGC0000010 | Aflatoxin\_biosynthetic\_gene\_cluster | 31 | 45.3846153846 | 52 | 6.5e-07 |
| AAS90051.1 | AflR | BGC0000009 | Aflatoxin\_biosynthetic\_gene\_cluster | 34 | 42.6923076923 | 50 | 4.2e-06 |
